# Reducing time from presentation to diagnosis of scaphoid fractures with cone beam CT: a before-and-after study

**DOI:** 10.1136/emermed-2024-214271

**Published:** 2025-10-12

**Authors:** Leah Flanagan, Sinead Loughran, Bibi Ayesha Bassa, Grainne Colgan, Etimbuk Umana, Vinny Ramiah, Michael Mara

**Affiliations:** 1 Emergency Department, Mater Misericordiae University Hospital, Dublin, Ireland; 2 Emergency Medicine, Mater Misericordiae University Hospital, Dublin, Ireland; 3 Royal College of Surgeons in Ireland, Dublin, Ireland; 4 Trauma and Orthopaedic Surgery, Mater Misericordiae University Hospital, Dublin, Ireland

**Keywords:** emergency department, extremity, Computed Tomography, Emergency Medicine

## Abstract

**Background:**

Scaphoid fractures comprise approximately 50–70% of carpal bone fractures but can be difficult to detect on initial plain film radiographs. A delayed diagnosis can lead to a high rate of non-union, avascular necrosis and Complex Regional Pain Syndrome. Current literature supports cone beam CT (CBCT) (within 10–14 days) as an effective method for diagnosing scaphoid fractures. We implemented an early outpatient CBCT pathway, prior to specialist review, with the aim to increase the proportion of patients with suspected scaphoid fracture undergoing CBCT within 7 days.

**Methods:**

We designed an ambulatory pathway for suspected scaphoid fractures in the Emergency Department (ED) in which outpatient CBCT was requested by emergency medicine clinicians. A retrospective audit of current management of these patients was performed between 1 August 2022 and 31 October 2022 (prepathway period). A list of patients who underwent CBCT performed for the indication ‘suspected scaphoid or carpal bone fracture’ in the hospital was obtained and screened. Implementation of the pathway took place in February 2023 and was reviewed by continuous audit monitoring from 1 March 2023 to 31 May 2023 (postpathway period).

**Results:**

Prepathway implementation, 54 patients underwent CBCT. Following implementation of our pathway, the number of CBCTs performed in the hospital for this clinical indication increased to 111 (postpathway). The proportion of patients undergoing CBCT within 7 days increased from 11.1% (6/54) to 91.8% (102/111) (p<0.000). There was a 71.9% reduction in fracture clinic attendances (50/54 (92.6%) prepathway and 23/111 (22.5%) post pathway (p<0.000).

**Conclusion:**

We successfully implemented an ambulatory pathway for suspected scaphoid fractures in the ED that significantly increased the proportion of patients with suspected scaphoid fractures undergoing early (<7 days) CBCT and definitive care.

**WHAT IS ALREADY KNOWN ON THIS TOPIC**

- Scaphoid fractures represent a significant concern for clinicians in the emergency department as they are often missed on initial plain film radiography and on follow-up imaging. Current guidelines recommend early MRI, but this practice is limited by availability and cost, leading to delays to diagnosis and intervention as well as prolonged unnecessary immobilisation. Early cone beam CT (CBCT) (within 10–14 days) is recognised as an effective alternative method for diagnosing scaphoid fractures.

**WHAT THIS STUDY ADDS**

- This study evaluates a new ambulatory pathway for suspected scaphoid fractures in the emergency department. This pathway uses CBCT for the diagnosis of suspected scaphoid fractures. The use of this pathway has demonstrated an increase in the proportion of patients undergoing CBCT within 7 days of initial presentation and its potential to decrease time to definitive imaging in patients with suspected scaphoid fractures.

**HOW THIS STUDY MIGHT AFFECT RESEARCH, POLICY OR PRACTICE**

- The findings of this study suggest that integrating a dedicated CBCT pathway into the outpatient management of patients with suspected scaphoid fractures can improve the proportion of patients undergoing early CBCT and expedite patient care. CBCT should be considered as an alternative to MRI in the investigation of patients with suspected scaphoid fractures.

## Introduction

 Acute wrist injuries are a common presentation to the emergency department (ED). Scaphoid fractures account for 60–80% of all carpal bone fractures but present a diagnostic challenge.^1–3^ Missed scaphoid fractures are associated with significant patient morbidity including complications such as avascular necrosis, non-union, carpal instability and degenerative changes, which can be life-altering.^4^ Conversely, empiric immobilisation of all patients with acute wrist injuries with negative initial plain film radiographs can lead to stiffness, increase time to rehabilitation and present economic consequences due to absence from work.^4 5^ Considering that the incidence of true positive scaphoid fractures in those with acute wrist injuries has been reported as approximately 16%, this practice can lead to a significant number of inappropriate and unnecessary immobilisations.^6 7^

The timely diagnosis and management of scaphoid fractures is crucial. Current guidance is conflicted with respect to recommended timing and imaging modalities. Cross-sectional surveys have demonstrated significant variation across centres in their investigation pathways and the imaging modalities employed both in the UK and internationally.^8 9^ Initial plain film radiographs are the primary imaging modality utilised at the point of presentation; approximately 75% of scaphoid fractures are identified on initial plain film radiographs.^2 6 10^ Repeating plain film radiographs within 7–10 days can increase the sensitivity of detecting a scaphoid fracture but rates remain low. Guidelines from the Royal College of Emergency Medicine, the UK National Institute of Care and Health Excellence and the British Society for Surgery of the Hand, recognise MRI as the gold standard for diagnosis.^11–13^ However, MRI can be costly and resource-intensive. Access can also be limited depending on the institution, especially within the time constraints recommended in clinical guidelines.^8 14^

CT has been proposed as an alternative to MRI with comparable sensitivity and specificity but involves considerable radiation exposure.^7,15^ Dutta *et al* demonstrated that early and efficient use of both CT and Virtual Fracture Assessment Clinic (VFAC) reduces the number of face-to-face clinic appointments and leads to a definitive plan and diagnosis for patients sooner.^15^ Cone beam CT (CBCT) is a smaller, conical unit using less radiation that was previously used in maxillofacial surgery.^16^ It has demonstrated promising results in the realm of bone and joint trauma after having previously been used in oral and maxillofacial surgery.^16–18^ When compared with plain film imaging, CBCT has since been shown to have a higher specificity, sensitivity, positive and negative predictive value for identifying scaphoid fractures.^10 19–23^ Accessing such imaging at an earlier time point could decrease time to definitive diagnosis. Most studies/quality improvement projects have reported incorporation of MRI into the pathway with very little evidence available for CBCT.^24 25^

Given the potential demonstrated in the literature for CBCT, we designed and implemented a before-and-after uncontrolled interventional study in order to increase the proportion of patients with suspected scaphoid fracture undergoing CBCT within a time-appropriate window and expedite patient care.

## Methods

### Study design

This was a prospective interventional before-and-after study with pre-post study methodology, occurring between 1 August 2022 and 31 May 2023. We conducted a 3-month before and after analysis comparing a newly designed CBCT pathway with the previous care stream of referring all suspected scaphoid fractures to an outpatient orthopaedic fracture clinic.

Exclusion criteria included patients with a confirmed fracture on the initial plain film X-rays and/or patients with atraumatic wrist pain. We excluded CBCT orders if the indication was for an alternative reason other than for a query scaphoid or carpal bone fracture, if notes were not available on the hospital electronic patient information system (ePIS) and instances of duplicate requests for the same patient.

### Setting

This pathway was implemented in a level 4 teaching institution and a trauma-receiving hospital for the central trauma network in the inner city of Dublin, Ireland, with 997 hospital beds. The ED has an annual attendance of 80 000 patients. It is also affiliated with a Minor Injuries Unit (MIU) with an independent attendance of 20 000 patients per annum. The trauma and orthopaedic department employs two specialist upper limb and hand surgeons. A CBCT scanner was already available in the hospital prior to the pathway implementation, which was being used for preoperative planning of bone and small joint injuries and diagnosis of occult radiocarpal fractures.^23^

Prior to the implementation of the CBCT pathway, neither the ED nor MIU had a standardised pathway for the management of suspected scaphoid fractures. All patients with suspected scaphoid fractures with initial negative plain-film radiographs were referred for follow-up assessment and/or CBCT in the outpatient orthopaedic fracture clinic, with wait times up to 14 days. This practice led to delays in accessing clinics for all orthopaedic patients, but also meant that the clinics were often busy and patients would wait long hours to be seen by the appropriate specialist. Importantly, CBCT images would be reviewed and patient disposition decided within the clinic time-frame, without access to formal radiology reports.

### Design and implementation of scaphoid fracture CBCT pathway

**Figure 1:**
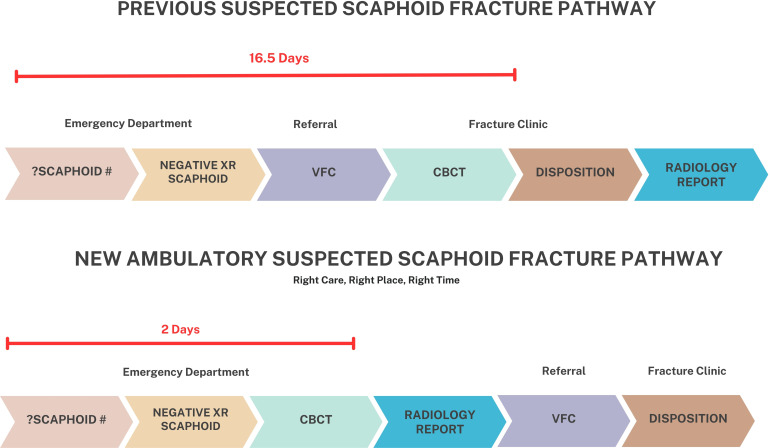
Value-stream maps before and after implementation of CBCT referral pathway for suspected scaphoid fractures. CBCT, Cone Beam CT; XR, X-Ray; VFC, Virtual Fracture Clinic.

Following the findings of the initial audit (tables 1–3) and discussion with stakeholders in emergency medicine (EM), radiology, trauma and orthopaedics and advanced nurse practitioners (ANPs) in the MIU (online supplemental Figure S1), we conducted a Strength, Weakness, Opportunity and Threat analysis of the proposed project (online supplemental Table 1). In addition, we engaged in ‘lean thinking’ for the purposes of this project, as we recognised that identifying and eliminating unnecessary steps in the patient’s journey with continuous pathway review would be the most effective method of transforming our pathway. This mirrors the HSE Sláintecare model of ‘right care, right place, right time’.^26^ We employed lean methodology by devising a value-stream map (figure 1) of the previous pathway that was in place for the investigation and management of patients with suspected scaphoid fractures.

**Table 1:** Demographics

	Prepathway audit (August–October 2022) (n=54)		Post-Pathway implementation (March–May 2023) (n=111)		P value
Male N(%) Female N(%)	27 (50.0%) 27 (50.0%)		52 (46.8%) 59 (53.2%)		0.742
Age N(%)	16–30 years 31–50 years 51–65 years >65 years	20 (37.0%) 21 (38.9%) 10 (18.5%) 3 (5.6%)	16-30 years 31-50 years 51-65 years >65 years	38 (34.2%) 44 (39.6%) 23 (20.7%) 6 (5.4%)	0.502
Mechanism of injury N(%)	Alleged assault FOOSH Sports injury Cycling E-scooter/e-bike RTA Other	3 (5.6%) 30 (55.6%) 11 (20.4%) 3 (5.6%) 3 (5.6%) 1 (1.9%) 3 (5.6%)	Alleged assault FOOSH Sports injury Cycling E-scooter/e-bike RTA Other	4 (3.6%) 61 (55.0%) 16 (14.4%) 11 (9.9%) 2 (1.8%) 1 (0.9%) 16 (14.4%)	0.321
Delay to present to hospital from date of injury >7 days N(%)	13 (24.1%)		14 (12.6%)		0.074
Pathology identified on CBCT N(%)	29 (53.7%)		54 (51.4%)		0.620

CBCT, cone beam CT; FOOSH, fall on out-stretched hand; RTA, Road Traffic Accident.

**Table 2:** Outcome measures

	Prepathway audit (August–October 2022) n=54	Post-Pathway implementation (March–May 2023) n=111	Difference in proportion	P value
Proportion of patients receiving CBCT within 7 days N(%)	6/54 (11.1%)	102/111 (91.8%)	80.7% (95% CI 70.3% to 91.7%)	<0.000
Proportion of patients seen in fracture clinic (N%)	50/54 (92.6%)	25/111 (22.5%)*	- 70.1% (95% CI −82.0% to −58.2%)	<0.000

* 48 (43.24%) referred to VFC for review.

CBCT, cone beam CT; VFC, Virtual Fracture Clinic.

**Table 3:** Key time intervals

	Prepathway audit (August–October 2022) n=54	Postpathway implementation (March–May 2023) n=111	P value*
Median time from presentation to CBCT (days)(CI)	16.5 (CI 15.0 to 19.0)	2.0 (CI 2.0 to 3.0)	<0.001
Median time from presentation to radiology report/ diagnosis (days)(CI)	18.0 (CI 16.0 to 20.0)	3.0 (CI 2.0 to 4.0)	<0.001
Median time from presentation to orthopaedic review/decision (days)(CI)	19.5 (CI 17.3 to 23.0)	16.0 (CI 13.1 to 18.9)	0.005

* Mann-Whitney test was used as the test of significance between before and after groups. T-test used for approximate point difference and CI between means of before and after groups.

CBCT, cone beam CT.

**Figure 2:**
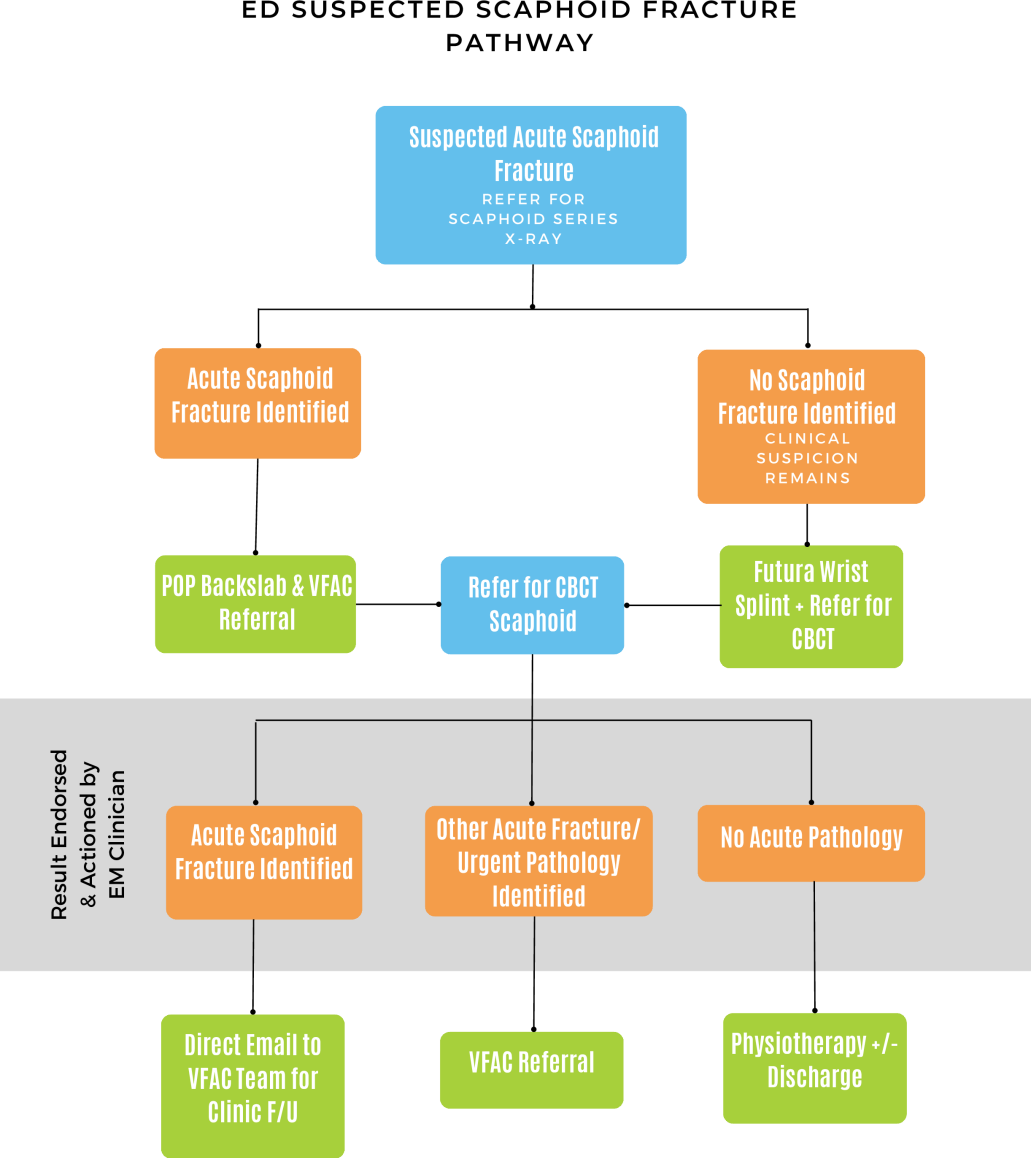
Flow Diagram of CBCT Referral Pathway for Suspected Scaphoid Fractures. CBCT, cone beam CT; ED, emergency department; EM, emergency medicine; VFAC, Virtual Fracture Assessment Clinic, POP, plaster of paris; F/U, follow-up.

Following an iterative process, we designed a direct referral pathway for patients presenting to the ED or MIU with acute wrist injuries concerning for acute scaphoid fracture (figure 2). Patients with clinical signs of scaphoid fracture but normal initial plain film radiographs were referred for CBCT by either an ANP or emergency clinician (foundation year 2 or above). These clinical signs were advised on the ‘standard operating procedure’ available on the ePIS.

A CBCT was ordered through the ePIS and a referral document was saved to generate an automatic worklist accessible by the radiographers, the radiology and EM team. The patient would be discharged in a Futura wrist splint from the ED or MIU. The radiology department’s administrative staff subsequently provided the patient with an outpatient appointment for CBCT at the next available time slot during working hours.

On completion of CBCT, the radiology team would report the findings, and this report would be made available to the EM consultant for review. Review of CBCT reports was the responsibility of one dedicated EM consultant in order to ensure adherence to the pathway in the early stages. In instances of no significant pathology, patients would be contacted by the EM consultant, counselled and advised immediate removal of splint and/or referral to physiotherapy if there was ongoing pain. If a fracture was identified, a referral to the VFAC would then be made, where the orthopaedic consultant would triage the patient for either physiotherapy or to the next appropriate clinic appointment.

Following implementation of the pathway in February 2023, we reviewed the pathway on a monthly basis for a further 3-month period between 1 March 2023 and 31 May 2023 using the same methodology as described above.

### Outcomes

Our primary outcome was ‘the proportion of patients undergoing CBCT within 7 days of presentation.’ Current guidelines suggest repeating imaging within 10–14 days of presentation.^8 11 12^ Following consultation with our specialist hand and upper limb orthopaedic surgeons, the decision was made to further reduce our goal to 7 days, which was felt to be realistic and achievable.

Secondary outcomes included reducing fracture clinic attendances by 50% for suspected scaphoid fractures, as well as achieving a 90% compliance rate with the new CBCT pathway.

### Data collection and analysis

Data were collected by our local radiography team for all CBCT wrist/hand examinations performed during the study period, screening for ‘suspected scaphoid fracture’ as the indication. The hospital ePIS was used to extract the patient’s demographics (age, self-reported sex), timing of the injury and presentation, time of CBCT and follow-up in the outpatient fracture clinic. Extracted data were stored using anonymous identifiers for patients on Microsoft Excel.

### Statistical analysis

Following MDT stakeholder engagement and based on the initial audit showing 11.1% of patients receiving CBCT within 7 days, we agreed that an increase to 50% was achievable and feasible. With an 80% power and 5% significance level, the study would require 42 patients (21 before and 21 after the intervention). Our initial audit identified 54 patients who underwent CBCT, suggesting we would meet or exceed the required sample size threshold.

Descriptive statistics were used to summarise baseline demographic characteristics. Categorical variables were reported as percentages and absolute counts. Continuous variables were shown as medians and IQR. For comparisons, where appropriate, Mann-Whitney test or two-sample t-test was used for continuous variables, and Fisher’s exact test or χ^2^ test was used for categorical variables. Statistical analyses were performed using STATA V.18.5.

### Bias

Several potential sources of bias identified by the authorship team included selection bias, detection bias and temporal bias. Selection bias may exist due to the significant increase in CBCT utilisation between periods. Detection bias may arise from the transition of CBCT ordering from primarily orthopaedic to EM clinicians, potentially reflecting different thresholds for investigation. The short periods of analysis preintervention and postintervention may introduce temporal bias. Information bias may result from retrospective data collection in the preintervention period. Additionally, performance bias could exist as staff were aware of pathway implementation and monitoring.

## Results

During the study period, 156 patients underwent plain film X-rays for suspected scaphoid fracture in the prepathway period (August–October 2022) and 147 patients in the postpathway period (March–May 2023). The number of patients that underwent CBCT increased from 54 patients (August–October 2022/pre-pathway) to 111 patients in the post-pathway period (March-May 2023/Post-pathway). Comparing pre-pathway to post-pathway implementation (table 1), there was no statistically significant difference with respect to age (p=0.502), gender (p=0.742), mechanism of injury (p=0.321) or presentation delays (p=0.074) of the populations.

**Figure 3:**
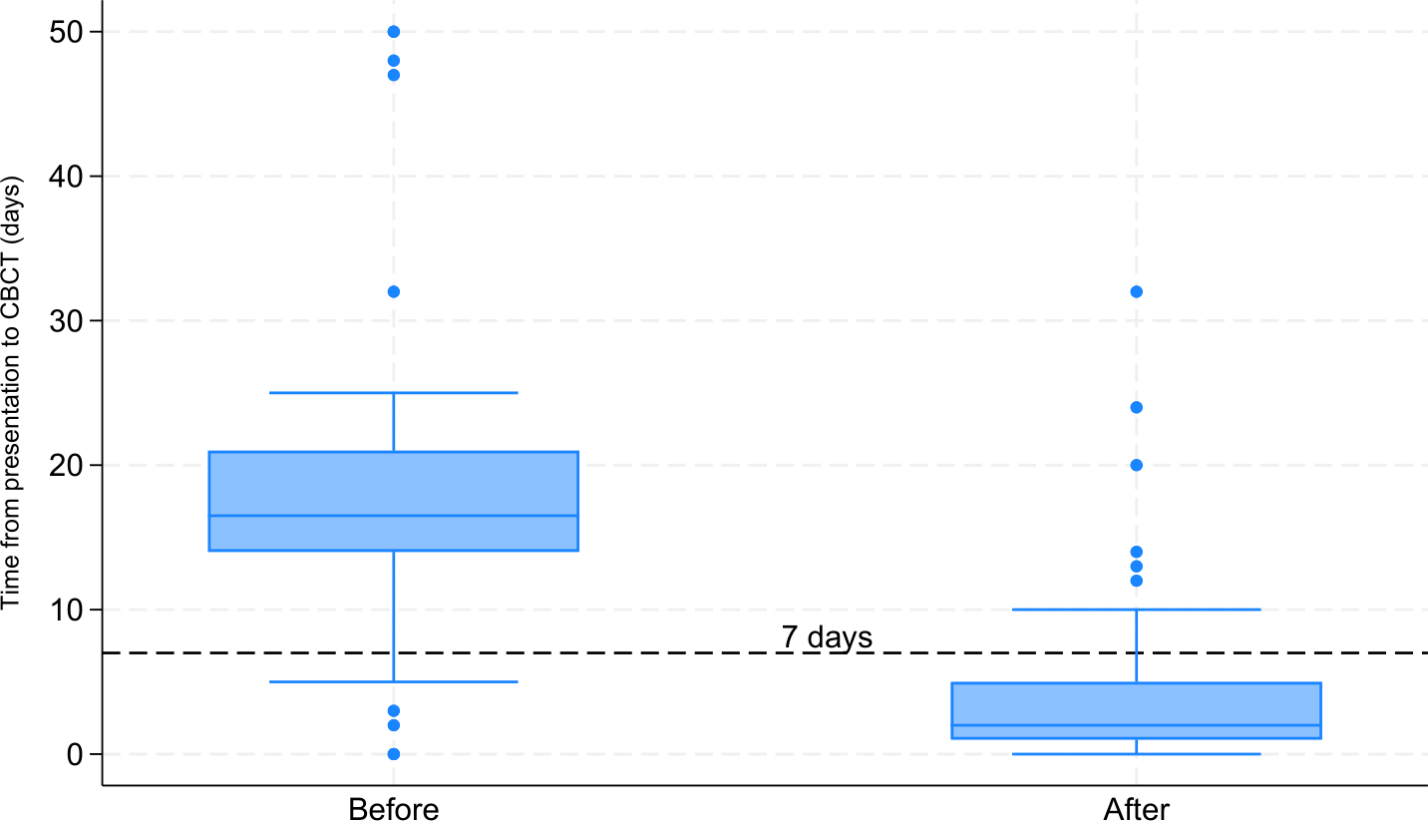
Reduction in time from presentation to diagnosis (CBCT) after pathway implementation. CBCT, cone beam CT.

There was an increase in the proportion of patients undergoing CBCT within 7 days of presentation from 11.1% in the prepathway group to 91.8% in the postpathway group (p<0.000) (figure 3). There was a reduction in fracture clinic attendances from 92.6% to 22.5% (p<0.000) (table 2). There was a compliance rate of 94.6% after pathway implementation. The median time from injury to other lean time intervals is outlined in table 3.

Of interest, prior to the pathway implementation, 90.7% of the CBCTs were ordered by the orthopaedic teams, which decreased to 3.6% postpathway. Additionally, 45.5% of patients had alternative fractures identified on their CBCT which had not been identified on the initial plain film imaging.

Of the 165 patients included in the analysis, 10 (6.1%) had acute scaphoid fractures. Of the 10 patients with acute scaphoid fractures, three (30.0%) underwent operative intervention. Online supplemental Table S2 outlines the number of other positive findings identified on CBCT.

## Discussion

This EM ambulatory pathway for suspected scaphoid fractures reduced diagnostic times and fracture clinic attendances. There was an 80.7% increase in the proportion of patients undergoing CBCT within 7 days (median 16.5–2.0 days), exceeding our target of 7 days, as well as current recommendations of 10–14 days.^8 11 12^ This proposed pathway, alongside other notable studies, demonstrates the ability of CBCT to provide earlier access to definitive imaging in patients with suspected scaphoid fractures, particularly in settings with limited access to MRI.^15^

### Strengths and weaknesses

Institutions in settings similar to our own have implemented local scaphoid pathways using MRI as an alternative imaging modality.^25^ The authors, however, acknowledge that early access to imaging in the form of MRI may not be achievable in other settings. While MRI remains the gold standard for scaphoid fracture diagnosis, this CBCT pathway offers a safe, practical and accessible alternative.

The high compliance rate of 94.6% suggests ease of integration of this pathway into clinical practice. This may be attributed to our comprehensive stakeholder engagement, clear protocol guidance and the ease of access to the pathway using the ePIS. These findings suggest that the pragmatic nature of this intervention, which does not go against the normal workflow, may increase the likelihood of long-term sustainability.

Multidisciplinary collaboration was key to the successful functioning of this pathway. Collaborative work from teams from EM, radiology, trauma and orthopaedics and our ANP team, as well as continuous audit monitoring, was key to the project’s success and strengthened institutional validity.

We acknowledge several limitations to our study. As a single-centre, uncontrolled before-and-after study, our findings may not be directly generalisable to other settings, particularly those without existing CBCT facilities and appropriate radiology and/or orthopaedic infrastructure. The 3-month preintervention and postintervention periods were relatively short and may not accurately reflect seasonal variations in presentation patterns.

In addition, over the 3-month period, there was a 205.6% increase in the number of patients undergoing CBCT for suspected scaphoid fractures. This would suggest that there was a lower threshold to image patients, given ease of access to the pathway. However, the rate of detection of pathology remained mostly unchanged (53.7% prior to the implementation of the pathway and 51.4% after). Although these findings suggest that the number of increased scans is appropriate and proportional to the number of presentations, the workload burden on the radiology department was not assessed. A follow-on feasibility study would be useful to assess the sustainability of our pathway and identify any implementation challenges over time. While we demonstrated improvements in process measures, our study did not assess patient-reported outcomes, satisfaction scores, evaluation of their experience or long-term clinical outcomes. Future studies should incorporate these measures to provide a more comprehensive evaluation of the pathway’s impact.

### Implications

The identification of alternative pathology in 58.8% (n=97) of cases highlights the potential diagnostic value of CBCT over plain radiographs. Although none of these alternative findings required surgical management, 13.3% (n=10) required longer periods of immobilisation in casts. This finding supports the use of CBCT as an early imaging modality in suspected carpal bone fractures, as these alternative injuries may be missed. The use of a virtual pathway for suspected carpal fractures that educates patients regarding their injury and encourages them to initiate follow-up may be appropriate in such instances.^15 24 27^

## Conclusion

In conclusion, we successfully implemented an EM ambulatory pathway for patients with suspected scaphoid fractures using CBCT. This pathway significantly increased the proportion of patients undergoing time-appropriate diagnostic imaging from 11.1% to 91.8%, in line with current international standards. This is a potentially safe and practical means to improve patient experience of acute wrist injuries and reduce orthopaedic fracture clinic attendances.

## Supplementary material

10.1136/emermed-2024-214271 online supplemental file 1

10.1136/emermed-2024-214271 online supplemental file 2

10.1136/emermed-2024-214271 online supplemental file 3

## Data Availability

All data relevant to the study are included in the article or uploaded as supplementary information.
