# Supplementary material for: Reducing time from presentation to diagnosis of scaphoid fractures with cone beam CT: a before-and-after study
Source: Emerg Med J. 2025 Oct 12;43(7):e214271. doi: 10.1136/emermed-2024-214271 (PMC13479200; doi:10.1136/emermed-2024-214271)
Supplement: Supplementary file 1 [file emermed-43-7-s001.pdf]

```
      ___  ____  ____  ____  ____(R)
     /__    /   ____/   /   ____/
    ___/   /   /___/   /   /___/
     Statistics/Data analysis

        User: Bibi Bassa
     Project: Leah
```

```
  ___  ____  ____  ____  ____ ®
 /__    /   ____/   /   ____/      StataNow 18.5
___/   /   /___/   /   /___/       SE—Standard Edition

 Statistics and Data Science       Copyright 1985-2023 StataCorp LLC
                                   StataCorp
                                   4905 Lakeway Drive
                                   College Station, Texas 77845 USA
                                   800-782-8272        https://www.stata.com
                                   979-696-4600        service@stata.com

Stata license: Unlimited-user network, expiring  9 Sep 2025
Serial number: 401809305307
  Licensed to: Bibi Bassa
               RCSI

Notes:
      1. Unicode is supported; see help unicode advice.
      2. Maximum number of variables is set to 5,000 but can be increased;
          see help set maxvar.
      3. New update available; type -update all-

1 . use "C:\Users\bibib\OneDrive\Desktop\Leah File.dta"

2 . ttest TimeppxtoCTCB, by(StudyGroup)

Two-sample t test with equal variances
```

Group	Obs	Mean	Std. err.	Std. dev.	[95% conf.	interval]
before	54	18.2963	1.462226	10.74513	15.36344	21.22915
after	111	3.486486	.4412893	4.649271	2.611955	4.361018
Combined	165	8.333333	.7800323	10.0197	6.793133	9.873534
diff		14.80981	1.197905		12.4444	17.17522

```
    diff = mean(before) - mean(after)                             t =  12.3631
H0: diff = 0                                     Degrees of freedom =      163

    Ha: diff < 0                 Ha: diff != 0                 Ha: diff > 0
 Pr(T < t) = 1.0000         Pr(|T| > |t|) = 0.0000          Pr(T > t) = 0.0000

3 . ttest Timeppxtoreport, by(StudyGroup)

Two-sample t test with equal variances
```

Group	Obs	Mean	Std. err.	Std. dev.	[95% conf.	interval]
before	54	19.14815	1.374218	10.0984	16.39182	21.90448
after	111	4.495495	.4479225	4.719156	3.607818	5.383173
Combined	165	9.290909	.7605877	9.769925	7.789102	10.79272
diff		14.65265	1.15173		12.37842	16.92689

```
    diff = mean(before) - mean(after)                             t =  12.7223
H0: diff = 0                                     Degrees of freedom =      163

    Ha: diff < 0                 Ha: diff != 0                 Ha: diff > 0
 Pr(T < t) = 1.0000         Pr(|T| > |t|) = 0.0000          Pr(T > t) = 0.0000
```

4 . ttest TimeppxtoorthoRV, by(StudyGroup)

Two-sample t test with equal variances

Group	Obs	Mean	Std. err.	Std. dev.	[95% conf.	interval]
before	**54**	**23.48148**	**1.55961**	**11.46075**	**20.3533**	**26.60966**
after	**25**	**16**	**1.354006**	**6.770032**	**13.20547**	**18.79453**
Combined	**79**	**21.11392**	**1.209745**	**10.75244**	**18.70551**	**23.52234**
diff		**7.481481**	**2.475191**		**2.552746**	**12.41022**

diff = mean(**before**) - mean(**after**) t = **3.0226**
H0: diff = 0 Degrees of freedom = **77**

Ha: diff < 0 Ha: diff != 0 Ha: diff > 0
Pr(T < t) = **0.9983** Pr(|T| > |t|) = **0.0034** Pr(T > t) = **0.0017**

5 . ttest TimeppxtoorthoRV, by(StudyGroup)

Two-sample t test with equal variances

Group	Obs	Mean	Std. err.	Std. dev.	[95% conf.	interval]
before	**54**	**23.48148**	**1.55961**	**11.46075**	**20.3533**	**26.60966**
after	**25**	**16**	**1.354006**	**6.770032**	**13.20547**	**18.79453**
Combined	**79**	**21.11392**	**1.209745**	**10.75244**	**18.70551**	**23.52234**
diff		**7.481481**	**2.475191**		**2.552746**	**12.41022**

diff = mean(**before**) - mean(**after**) t = **3.0226**
H0: diff = 0 Degrees of freedom = **77**

Ha: diff < 0 Ha: diff != 0 Ha: diff > 0
Pr(T < t) = **0.9983** Pr(|T| > |t|) = **0.0034** Pr(T > t) = **0.0017**

6 .
