# Supplementary material for: Reducing time from presentation to diagnosis of scaphoid fractures with cone beam CT: a before-and-after study
Source: Emerg Med J. 2025 Oct 12;43(7):e214271. doi: 10.1136/emermed-2024-214271 (PMC13479200; doi:10.1136/emermed-2024-214271)
Supplement: Supplementary file 3 [file emermed-43-7-s002.docx]

# Supplementary Material EMJ

**Supplementary Figure S1: Stakeholder Analysis**

| **Supplementary Table S1: SWOT Analysis** | |
| --- | --- |
| **Strengths** | **Weaknesses** |
| - Experienced consultant colleagues across a variety of specialties including emergency medicine, radiology and orthopaedic hand surgery - Implementation by a number of our Advanced Nurse Practitioners who saw these patients in our off-site Minor Injuries Unit (MIU) - Access to CBCT within our facility | - Only one EM consultant available to manage the worklist and review CBCT reports and implement change - Shortages in staffing in radiology and radiography with a suspected increase in scan quantities - Increased workload burden on an already busy EM team |
| **Opportunities** | **Threat** |
| - IT system that allowed for integration of the pathway and development of a Scaphoid Pathway CBCT worklist that could be accessed by all stakeholder specialties - ‘Virtual Fracture Assessment Clinic’ pathway already in place whereby seamless referral of patients could take place and consultant orthopaedic surgeons could review case history and CBCT images and organise the most appropriate, rapid follow up in Fracture Clinic | - ED often had a high turnover of staff or increased numbers of locum NCHDs which would prove difficult to increase awareness about the pathway - Possible misuse of the pathway in patients with atraumatic, chronic hand and wrist pain in order to access earlier diagnostic imaging |

| **Supplementary Table S2: CT Cone Beam Report Findings & Scaphoid Fracture Management** | | | | |
| --- | --- | --- | --- | --- |
|  |  | **Pre-Pathway**  (Aug-Oct 2022)  N=54  N (%) | **Post-Pathway** (Mar-May 2023)  N=111  N (%) | P Value |
| Injuries N(%) | **1.No Acute Pathology**  **2.Scaphoid**  **3.Other pathology**  Capitate  Distal Radius  Distal Ulna/Ulnar Styloid  Hamate  Lunate  Metacarpal  Other  Pisiform  Scapholunate Ligament  Trapezium  Trapezoid  Triquetrum | 25 (46.3%)  5 (9.3%)  24  0 (0.0%)  6 (11.1%)  1 (1.9%)  2 (3.7%)  5 (9.3%)  3 (5.6%)  4 (7.4%)  2 (3.7%)  2 (3.7%)  5 (9.7%)  1 (1.9%)  3 (5.6%) | 54 (48.7%)  5 (4.5%)  52  6 (5.4%)  16 (14.4%)  1 (0.9%)  5 (4.5%)  5 (4.0%)  4 (3.6%)  2 (1.8%)  1 (0.9%)  4 (3.6%)  12 (10.8%)  3 (2.7%)  10 (9.0%) | 0.492 |
| Scaphoid Fracture Management | Total (N)  Operative  Non-operative  Proceeded to MRI | 5 (9.3%)  1 (20.0%)  3 (60.0%)  1 (20.0%) | 5 (4.5%)  2 (40.0%)  2 (40.0%)  1 (20.0%) | 1.0 |
| **Some patients had more than one pathological finding identified on CBCT* | | | | |
